# Betahistine add-on therapy for treatment of subjects with posterior benign paroxysmal positional vertigo: a randomized controlled trial

**DOI:** 10.1016/j.bjorl.2020.07.011

**Published:** 2020-09-12

**Authors:** Ibrahim Sayin, Recep Haydar Koç, Dastan Temirbekov, Selcuk Gunes, Musa Cirak, Zahide Mine Yazici

**Affiliations:** a Bakırköy Teaching and Research Hospital, Department of Otolaryngology Head and Neck Surgery, Istanbul, Turkey; b Istanbul Aydın University, Medical Park Florya Hospital, Department of Otorhinolaryngology, Istanbul, Turkey; c Bakırköy Teaching and Research Hospital, Department of Neurosurgery, Istanbul, Turkey

**Keywords:** Betahistine, Vertigo, Dizziness, Epley maneuver, Quality of life

## Abstract

**Introduction:**

Benign paroxysmal positional vertigo is a common vestibular disorder that accounts for one fifth of hospital admissions due to vertigo, although it is commonly undiagnosed.

**Objective:**

To evaluate the effects of betahistine add-on therapy in the treatment of subjects with posterior benign paroxysmal positional vertigo.

**Methods:**

This randomized controlled study was conducted in a population of 100 subjects with posterior benign paroxysmal positional vertigo. Subjects were divided into the Epley maneuver + betahistine group (group A) and Epley maneuver only (group B) group. Subjects were evaluated before and 1-week after the maneuver using a visual analog scale and dizziness handicap inventory

**Results:**

One hundred subjects completed the study protocol. The Epley maneuver had an overall success rate of 95% (96% in group A; 94% in group B, *p* =  0.024). Groups A and B had similar baseline visual analog scale scores (6.98 ± 2.133 and 6.27 ± 2.148, respectively, *p* = 0.100). After treatment, the visual analog scale score was significantly lower in both groups, and was significantly lower in group A than group B (0.74 ± 0.853 vs. 1.92 ± 1.288, respectively, *p* = 0.000). The change in visual analog scale score after treatment compared to baseline was also significantly greater in group A than group B (6.24 ± 2.01 vs. 4.34 ± 2.32, respectively, *p* = 0.000). The baseline dizziness handicap inventory values were also similar in groups A and B (55.60 ± 22.732 vs. 45.59 ± 17.049, respectively, *p* = 0.028). After treatment, they were significantly lower in both groups. The change in score after treatment compared to baseline was also significantly greater in group A than group B (52.44 ± 21.42 vs. 35.71 ± 13.51, respectively, *p* = 0.000).

**Conclusion:**

The Epley maneuver is effective for treatment of benign paroxysmal positional vertigo. Betahistine add-on treatment in posterior benign paroxysmal positional vertigo resulted in improvements in both visual analog scale score and dizziness handicap inventory.

## Introduction

Benign paroxysmal positional vertigo (BPPV) is a common vestibular disorder that accounts for one fifth of all admissions to hospital due to vertigo although it is commonly undiagnosed.^1^, ^2^ The most common form is idiopathic, and BPPV tends to occur at a higher rates in women than in men.^3^ The main mechanism underlying BPPV is accumulation of otoconia (calcium carbonate structures) in the lumen (canalolithiasis) or in the cupula (cupulolithiasis) of the semicircular canal, resulting in impaired fluid dynamics of the semicircular canal.^4^

Several factors have been investigated as possible causes of BPPV, including migraine, Ménière’s disease, idiopathic sudden sensorineural hearing loss, sleeping habits, osteoporosis/vitamin D insufficiency, hyperglycemia/diabetes mellitus, estrogen deficiency, neurological disorders, allergies, and others.^5^, ^6^ Regardless of the etiology and clinical picture, canalith repositioning maneuvers are the mainstay of BPPV treatment with level 1 evidence in treating BPPV.^7^ Although the methods used differ among physicians, BPPV canalith repositioning maneuvers have a high success rate. Quality of life and relief of dizziness are important issues during the healing process. A few medical treatment options are available, including administration of betahistine.

Betahistine acts as a weak agonist for H1 receptors and as an antagonist for H3 receptors.^8^ Betahistine is the main treatment option for Ménière’s disease. However, it is currently also used to treat various vestibular disorders as well as a number of other conditions, including tinnitus.^9^ A recent Cochrane review indicated a low quality of evidence in favor of betahistine for treating vertigo in a number of studies (pooled risk ratio for overall improvement, 1.30).^2^ This highlighted the need for additional clinical studies to understand the efficacy of betahistine in different types of vertigo. Previous studies have yielded conflicting results regarding the therapeutic use of betahistine in the acute phase of BPPV. The present randomized controlled trial was conducted to evaluate the effects of betahistine as add-on therapy in subjects treated with the Epley maneuver.

## Methods

This randomized controlled trial was performed at the Department of Otolaryngology Head and Neck Surgery Bakırköy Dr. Sadi Konuk Teaching and Research Hospital Hospital between April 2019 and January 2020. Ethical approval was obtained from the hospital ethics board (2019/164), and all subjects provided informed consent.

**Figure 1:**
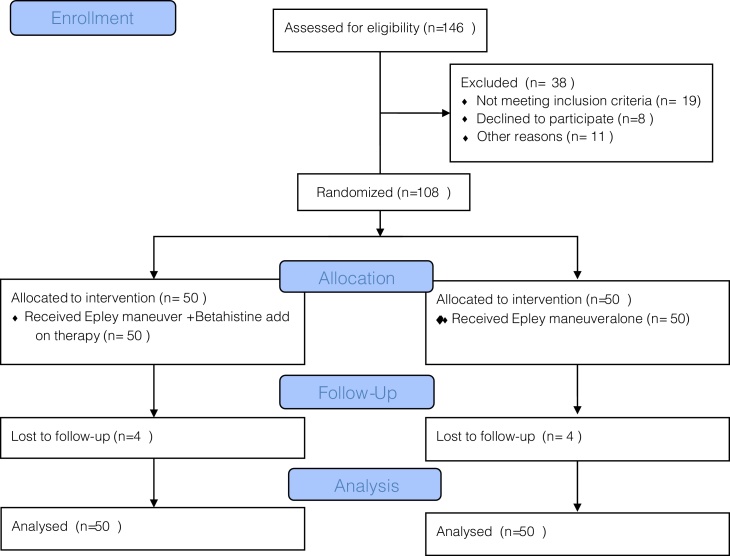
CONSORT flow diagram.

After power analysis, 146 subjects were evaluated for inclusion in the study. Thirty-eight subjects did not meet the inclusion criteria (Fig. 1). Hence, 108 subjects with posterior BPPV diagnosed via the Dix-Hallpike test were included. The following subjects were excluded: those with anterior BPPV, lateral BPPV, and age <18 years, those with a history of other vestibular pathologies (Ménière’s disease, vestibular neuritis), neurological disorders (e.g., migraine, CVO), psychiatric disorders (depression), and/or concomitant cardiovascular or cerebrovascular diseases, a history of sudden hearing loss or previous ear operations (e.g., tympanoplasty, mastoidectomy) the use of antivertiginous drugs, antihistamines, benzodiazepines, calcium channel blockers, and/or thiazide diuretics, and those with physical limitations that made them unsuitable for the the Epley maneuver (e.g., cervical stenosis, Down’s syndrome, rheumatological diseases, spinal cord injuries).

All subjects were diagnosed by the presence of rotary upbeat nystagmus provoked by the Dix-Hallpike maneuver. No additional diagnostic tools were used. After diagnosis, subjects were randomized into Group A (Epley maneuver + betahistine 24 mg, p.o., twice daily) and Group B (Epley maneuver only). The Epley maneuver was performed immediately after the Dix-Hallpike test. A Visual Analog Scale (VAS) and Dizziness Handicap Inventory (DHI) were evaluated before the maneuver. No positional restrictions were applied.

Subjects were evaluated 1-week after the maneuver. The Dix-Hallpike test was performed to verify the complete resolution of provoked nystagmus; if present, a second repositioning maneuver was performed, and subjects were evaluated with the DHI and VAS.

The DHI is the most widely used scale to assess the self-perceived handicapping effects imposed by diseases of the vestibular system.^10^ The patient answers “yes”, “sometimes”, or “no” to each question, and the strengths of the responses are indicated by numeric values of 0, 2, or 4. The questionnaire has 25 items, such that the total score can range from 0 to 100, with higher scores indicating a greater degree of handicap.

### Statistical analysis

Statistical analyses were performed using Number Cruncher Statistical System 2007 (NCSS, Kaysville, UT). After descriptive statistics (means, standard deviations, medians, and ranges) were calculated, the Student’s *t* test and χ^2^ test was used to evaluate the differences between groups according to age and sex. Assessments of whether patients with previous dizziness were distributed homogeneously between the groups with and without betahistine treatment were performed using the χ^2^ test. Student’s *t* test was used to compare pre- and post-treatment VAS scores between the groups with and without betahistine. The Mann-Whitney *U* test was used to compare pre- and post-treatment DHI scores. The Student’s *t* test and the Mann-Whitney U test were used to evaluate the differences in VAS scores and DHI scores after treatment compared to the respective baselines. In all analyses, *p* < 0.05 was taken to indicate statistical significance.

### Power analysis

Power analysis was performed using Gpower 3.1.9.2 (Heinrich-Heine-Universität Düsseldorf, HHU). Based on Güneri et al., the effect size was calculated as 0.3.^5^ For a power of 0.80 with an error margin of 0.05, 108 subjects were required for the study.

## Results

During the study, eight subjects were lost to followup. In total, 100 subjects completed the study protocol. The mean age of the total study population was 53.34 ± 15.33 years, and there were no significant differences in mean age between Group A and Group B (55.68 ± 14.534 vs. 50.96 ± 15.911 years, respectively, *p* = 0.126). Forty-two subjects were male. There were no significant differences in sex distribution between the two groups (*p* = 0.349). Forty-seven subjects had experienced a previous vertigo attack, and there were no significant differences in the distribution of previous vertigo attacks between the two groups (*p* = 0.270).

The Epley maneuver had an overall success rate in the total study population of 95% (n = 95), with rates of 96% (n = 48) in group A and 94% (n = 47) in Group B.

Groups A and B had similar baseline VAS scores (6.98 ± 2.133 vs. 6.27 ± 2.148, respectively, *p* = 0.100). After treatment, the VAS score was significantly lower in both groups, and was significantly lower in Group A than Group B (0.74 ± 0.853 vs. 1.92 ± 1.288, respectively, *p* = 0.000). The change in VAS score after treatment compared to the baseline was also significantly greater in Group A than Group B (6.24 ± 2.01 vs. 4.34 ± 2.32, respectively, *p* = 0.000).

The baseline DHI values were also similar in groups A and B (55.60 ± 22.732 vs. 45.59 ± 17.049, respectively, *p* = 0.028). After treatment, the DHI values were significantly lower in both groups. Group A had better VAS scores than Group B (3.16 ± 4.002 vs. 9.88 ± 8.616, respectively, *p* = 0.000). The change in DHI score after treatment compared to baseline was significantly greater in Group A than Group B (52.44 ± 21.42 vs. 35.71 ± 13.51, respectively, *p* = 0.000) (Table 1).

**Table 1:** Demographic parameters, Visual Analog Scale, and Dizziness Handicap Inventory in subjects given betahistine add-on therapy along with canalith repositioning maneuver for treatment of posterior benign paroxysmal positional vertigo.

		Total	Group A (n = 50)	Group B (n = 50)	*p*
**Age**	Mean ± SD	53.34 ± 15.33 (range 18 − 85)	55.68 ± 14.534 (range 29 − 82)	50.96 ± 15.911 (range 18 − 85)	0.126^a^
**Gender**	Male	42	23	19	0.349^b^
	Female	58	27	31	
**Previous vertigo attack**	Present	47 (47%)	21 (21%)	26 (26%)	0.270^b^
	Absent	53 (53%)	29 (29%)	24 (24%)	
**VAS**	Baseline	6.63 ± 2.160	6.98 ± 2.133	6.27 ± 2.148	0.100^a^
	Post-Epley maneuver	1.32 ± 1.236	0.74 ± .853	1.92 ± 1.288	0.000^a^

		**Difference**	**Difference**	**Difference**	
		5.30 ± 2.36	6.24 ± 2.01	4.34 ± 2.32	0.000c
		***p***	**0.000**	**0.000**	
**DHI**	Baseline	50.65 ± 20.640	55.60 ± 22.732	45.59 ± 17.049	0.028^d^
	Post-Epley maneuver	6.48 ± 7.467	3.16 ± 4.002	9.88 ± 8.616	0.000^d^

		**Difference**	**Difference**	**Difference**	
		44.16 ± 19.73	52.44 ± 21.42	35.71 ± 13.51	0.000^c^
		***p***	**0.000**	**0.000**	
**Epley maneuver success**		n = 95 (95%)	n = 48 (96%)	n = 47 (94%)	0.024^b^

Group A, Epley maneuver + Betahistine add on therapy; Group B, Epley maneuver alone.

VAS, Visual Analog Scale; DHI, Dizziness Handicap Inventory.

a Student *t* test.

b Chi Square.

c Linear regression analysis.

d Mann Whitney u test.

## Discussion

This randomized controlled study showed that betahistine add-on treatment resulted in better BPPV symptom improvement as verified by VAS and DHI. It did not affect the overall success rate of the canalith repositioning maneuver (95%).

A recent Cochrane review highlighted the need for rigorous future research regarding treatment of BPPV, including the use of strict inclusion criteria for subject selection, standard tools for diagnosis, power analyses, and reporting according to the CONSORT statement.^2^ Our study fulfilled most of these requirements.

Betahistine serves as a co-adjuvant treatment option for various disorders related to dizziness and vertigo and has been in clinical use since 1968. The level of evidence for betahistine is low for overall vertigo treatment. However, data in the literature support its use in various types of vertigo with a good safety profile.^8^, ^11^ Recent studies around the world have indicated that two thirds of subjects diagnosed with vertigo are prescribed betahistine, regardless of the etiology.^2^ The multicenter VIRTUOSO study, which included 305 subjects with vertigo, indicated that theclinical response to 48 mg daily betahistine was reported as good/excellent by 95.4% of patients and 94.4% of treating physicians in routine settings.^8^ The beneficial effects of betahistine on monthly vertigo attacks were evident during the 2-month treatment period but the effect was no longer seen by 2 months after cessation of treatment.

Betahistine acts as a histamine modulator, but its precise mechanism of action is not known. The main effect of betahistine is to improve the microcirculation of the inner ear with vasodilation. In the inner ear, the antagonistic effect of betahistine on H3 receptors is related to increased neurotransmitter release from nerve endings. In combination with the increased histamine levels from nerve endings, the direct agonistic effect of betahistine on H1 receptors expressed in blood vessels of the inner ear facilitates blood flow.^12^

In the cochlear region, betahistine exerts its effect via precapillary sphincters located on the stria vascularis.^9^ Relaxation of the precapillary sphincters results in a decrease in endolymphatic sac pressure, which can explain its efficiency in Ménière’s disease. Betahistine can also affect the firing of neurons in the vestibular nuclei. The effects of betahistine on the lateral and medial vestibular nuclei are dose dependent.^13^

Although betahistine has a good safety profile,^14^ limited data are available for its use in children, adolescents and the geriatric population.^9^ The typical dose is between 8 and 48 mg daily. Betahistine is contraindicated in pheochromocytoma, and care is required for its use in subjects with asthma and peptic ulcer. Frequent side effects include headache and mild gastrointestinal problems, but its safety profile has been reported to be similar to placebo. A recent Cochrane review reported betahistine and placebo to be associated with adverse events in 16% and 15% of subjects, respectively.^2^ To date, two serious adverse reactions have been reported with use of betahistine in more than 100 million people.^14^ These side effects were mild, dose-dependent, and temporary. In an open-label post-marketing survey, Benecke et al. reported an incidence of adverse events associated with betahistine of 2.4%, and drug discontinuation was needed in 17 (0.8%) subjects.^11^ In our study, no adverse events were reported and all subjects continued treatment.

Canalith repositioning maneuvers constitute the main treatment for BPPV.^7^ The clinical practice guidelines support the use of canalith repositioning maneuvers in treatment of BPPV.^15^ Several studies have demonstrated its efficacy. Prim-Espada et al. published a meta-analysis on the efficacy of the Epley maneuver in treatment of BPPV.^16^ Treated subjects had a 6.5 times greater chance of symptom improvement (OR = 6.52; 95% CI 4.17 − 10.20) and 5 times greater chance of a negative Dix-Hallpike test (OR = 5.19; 95% CI 2.41–11.17) than controls. Our study had an overall success rate of 95% for the canalith repositioning maneuver.

Although canalith repositioning maneuvers are the mainstay of treatment, options for symptom control are limited. Betahistine has been examined for symptom control in some studies. Previous studies have indicated its effectiveness in vestibular disorders, particularly in Ménière’s disease. Stombolieva and Angov suggested that betahistine improves blood flow in the inner ear, which may have a positive effect on postural instability.^4^

Previous reports on its use in BPPV have indicated that it leads to faster recovery and better symptom control. In a meta-analysis of seven studies, Della Pepa et al. reported that betahistine showed beneficial effects compared to placebo (OR = 3.52, relative risk 1.78).^17^ In a study of 90 subjects consisting of 30 treated with the Epley maneuver + betahistine, 30 treated with the Epley maneuver alone, and 30 treated with betahistine alone, Kaur and Shamanna reported a better response in the Epley maneuver + betahistine group.^3^ Subjects were evaluated at 1 and 4 weeks, and those receiving Epley maneuver plus betahistine experienced less recurrence and relapse. In the present study, we did not include a betahistine alone group as we believe that the main treatment modality for BPPV is the Epley maneuver, and that all subjects with BPPV should be given this treatment initially.

Cavaliere et al. compared the effects of the Semont maneuver and Brandt-Daroff maneuver with or without betahistine treatment in a total of 103 subjects divided into four groups. Subjects were evaluated on days 3, 7, 14, 30, 60, and 90. For both the liberatory and Brandt-Daroff maneuvers, betahistine add-on therapy resulted in faster recovery compared to the respective maneuver alone.^18^ This effect was also evident in older patients. The use of betahistine for over 3 months was reported to provide no additional benefit and use of betahistine did not affect the final improvement rate.

Güneri et al. conducted a double-blind randomized controlled clinical trial in a population of 72 subjects using four different vertigo symptom scales.^5^ The study group consisted of 24 subjects treated with the Epley maneuver alone, 24 who received Epley maneuver + betahistine (24 mg twice daily), and 24 who received Epley maneuver + placebo. The primary success rate of the Epley maneuver was reported as 86.2%. They also found that adding betahistine to the Epley maneuver improved symptom control. This effect was evident if the symptoms lasted for less than 1 month and if the subjects had hypertension, but no additional benefit was demonstrated for residual dizziness. Mira et al. conducted a double-blind, multicenter, placebo-controlled study in 144 subjects (81 with Ménière’s disease, 63 with BPPV). Use of betahistine was shown to decrease the frequency, intensity, and duration of vertigo attacks and to improve quality of life scores.^19^

Residual dizziness was reported at rates of 31% to 61% and could last for several weeks.^1^ Such dizziness can have an adverse effect on quality of life and increase the incidence of falls in the elderly. Jalali et al. conducted a randomized placebo-controlled trial in 117 subjects with posterior BPPV. After the Epley maneuver, subjects received 1 week of either betahistine, dimenhydrinate, or placebo treatment. The authors performed logistic regression analyses, and reported that subjects who received betahistine were 3.18 times more likely to have no residual dizziness compared to the placebo group.^1^ However, the beneficial effects of betahistine treatment were inversely correlated with age.

Other studies have indicated no efficacy of betahistine add-on treatment. Acar et al. conducted a prospective study in 100 subjects (25 received the Epley maneuver + betahistine, 25 received the Epley maneuver + trimetazidine, 25 received the Epley maneuver + Gingko Biloba, and 25 underwent the Epley maneuver alone) using the DHI questionnaire, and found no significant differences between groups after 3–5 days of treatment.^20^

The major limitation of the present study was the lack of betahistine-only and placebo groups. As canalith repositioning maneuvers are the mainstay of BPPV treatment, we did not include any untreated or placebo control groups for ethical reasons. We did not perform further long-term evaluation of residual dizziness as the repositioning maneuver used here results in excellent symptom control rates.

## Conclusion

Although canalith repositioning maneuvers remain the mainstay of treatment, betahistine add-on therapy resulted in better symptom control in subjects with posterior BPPV.

## Conflicts of interest

The authors declare no conflicts of interest.
